# Hydatid cyst of the heart with mitral valve stenosis; Case report

**DOI:** 10.1016/j.amsu.2019.11.018

**Published:** 2019-12-06

**Authors:** Alaa S. Bahjat, Ahamd Mohammad Sharif Tahir, Ayad Ahmad Mohammed

**Affiliations:** a Department of Surgery, College of Medicine University of Duhok, Duhok, Kurdistan Region, Iraq; b Duhok Directorate General of Health, Azadi Heart Center, Duhok City, Kurdistan Region, Iraq

**Keywords:** Hydatid cyst, Echinococcus granulosus, Mitral valve stenosis, Case report

## Abstract

Hydatid cyst of the heart is very rare, the left ventricle is the commonest site of myocardial involvement due to dominant left coronary vessels and thicker wall. Isolated cardiac involvement is extremely rare. Patients may be quite asymptomatic but the cyst may cause palpitation, dyspnea, chest pain, or when ruptured in to the cardiac or pericardial cavities may cause emergency presentations like anaphylactic reactions, sudden collapse due to pericardial tamponade or even sudden death.

A middle age female presented with exertional shortness of breath for 2 years. Echocardiography showed mitral valve stenosis. Computerized tomography scan of the chest showed a big complicated hydatid cyst arising from the wall of the right ventricle. Median sternotomy was done with excision of the hydatid cyst, and repair of mitral stenosis by commissurotomy. The patient received three cycles of albendazole for three months.

Surgery is the best options of treatment of cardiac hydatid disease, when the disease is affecting the pericardium complete excision may be possible, but when the myocardium is involved it may be difficult or even impossible to do complete excision, in this situation the cyst contents should be evacuated completely, preventing spillage is very mandatary to prevent recurrence. Care must be taken to avoid damage to the conductive system, the papillary muscles, the aortic and the mitral valves. Medical treatment with anthelminthic medications is used after surgery to reduce the recurrence rate.

## Highlights

• Hydatid cyst of the heart is a very rare clinical finding.
• It may be encountered during any other kinds of cardiac surgeries.
• The heart may be primarily affected of may be secondary to other organ involvement.

## 1 Introduction

Hydatid disease is cause when the human being is affecting by the embryonal stage of the parasite *Echinococcus granulosus*, this disease is endemic in certain parts of the world, such as the Mediterranean region, certain parts of the central Europe, some parts of new Zeeland [1].

The disease is transmitted by the fecal-oral route, and the eggs of the parasite hatch in the bowel and transmitted by the circulation. The disease primarily affects liver and the lungs, but many other organs could be affected. Hydatid cysts affects the heart through the coronary arteries. Isolated cardiac involvement is a unique clinical finding, the primarily affects the myocardium, it may rupture due to the motion of the heart of after trauma, the rupture could occur to the pericardial space and affects the pericardial layers which is almost always secondary to myocardial involvement or may rupture to the heart cavity causing embolization of the contents to various parts if the body [2].

The left ventricle is the commonest site of myocardial involvement due to dominant left coronary vessels and thicker wall [2,3].

Isolated cardiac involvement by hydatid disease is extremely rare and is estimated to occur in 0.02–2% of patients. Patients may be quite asymptomatic and discovered incidentally due to slow growth of the cyst, but the cyst may cause palpitation, dyspnea, chest pain, or when ruptured in to the cardiac or pericardial cavities may cause emergency presentations like anaphylactic reactions, sudden collapse due to pericardial tamponade or even sudden death [2,4].

The work in this case report has been reported in line with the SCARE 2018 criteria [5].

## 2 Patient information

A 58-year-old female presented with chronic history of exertional shortness of breath for 2 years. The patient had negative past medical and surgical histories, and the family history was positive for hypertension and diabetes mellitus.

The drug history, family for any genetic disorders, and psychosocial history were non relevant.

**Clinical findings:** The patient had normal general clinical examination with normal vital signs. Auscultation of the chest revealed a diastolic mummer.

**Diagnostic assessment:** Echocardiography showed mitral valve stenosis. Coronary angiography showed normal coronaries, but abnormal course of right coronary artery.

**Fig. 1:**
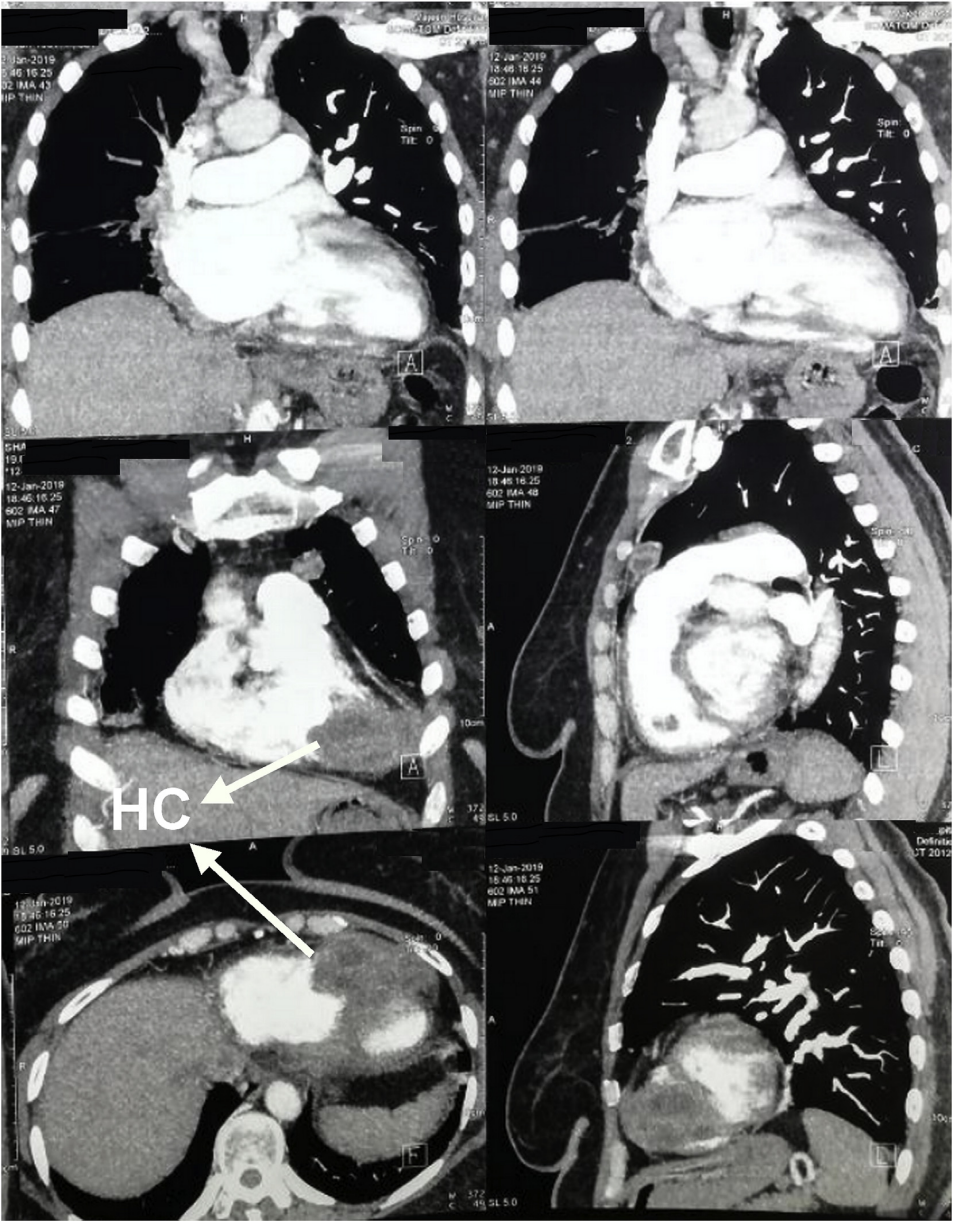
Computerized tomography scan of the chest showing a multi-loculated cystic lesion within the wall of the right ventricle, labelled with white arrow.

Patient started on medical therapy for 3 months for symptomatic mitral valve stenosis, but her symptoms continues. The echocardiography was repeated which detected an abnormal lesion in the right ventricle, further assessment was suggested. Computerized tomography scan of the chest was done and a big complicated hydatid cyst arising from the wall of the right ventricle was discovered. Fig. 1.

### 2.1 Therapeutic intervention

**Fig. 2:**
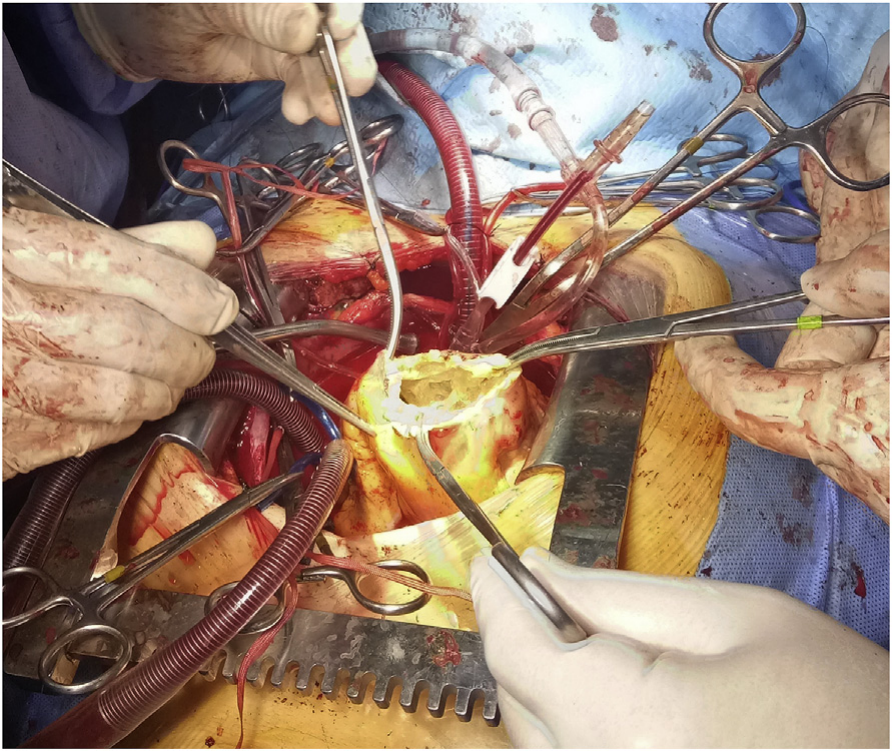
Cavity of hydatid cyst over the right ventricle, after removing the cyst contents.

**Fig. 3:**
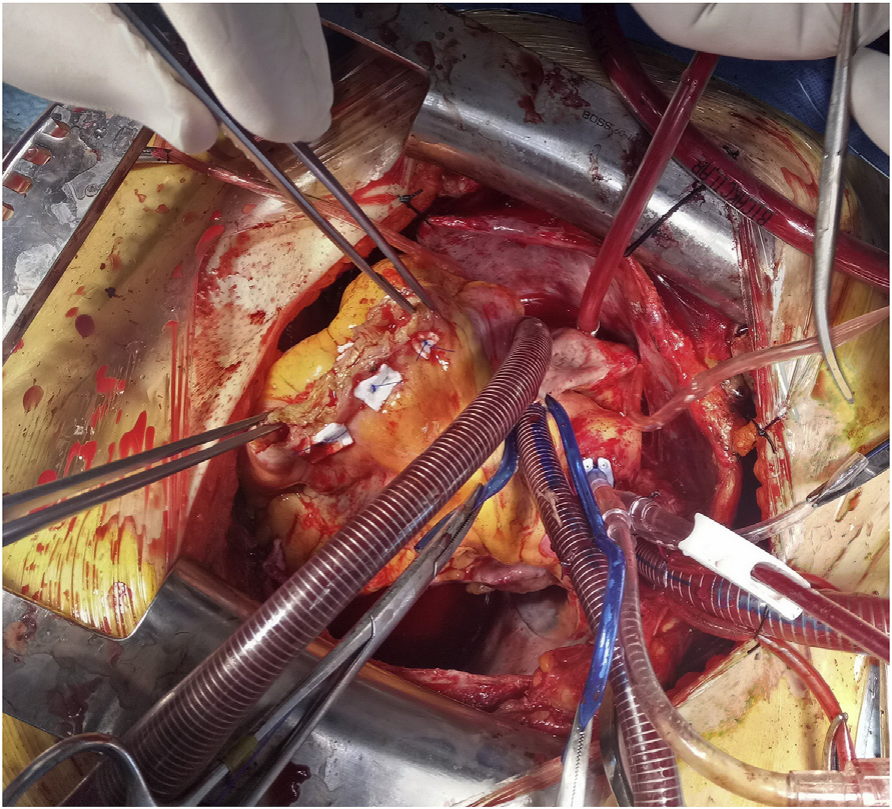
Closure of hydatid cyst cavity by pledgeted sutures.

Decision of surgery done, and the plan of surgery was to do median sternotomy, excision of the hydatid cyst, and repair of mitral stenosis by commissurotomy. Isolation of the hydatid cyst from the surgical field was done using surgical sponges soaked with chlorhexidine solution. The cyst was involving the muscular layer of the right ventricle, excision of the cyst was done and cavity of hydatid cyst was marsipulized and patient weaned from cardiopulmonary bypass. Fig. 2, Fig. 3.

The operation was performed by two cardiac surgeons who are specialized in the field of cardiac and vascular surgery.

### 2.2 Follow-up and outcomes

Postoperative echocardiography showed normal right ventricular function, mild valve mitral stenosis. Patient put on three cycles of albendazole for three months.

No specific post intervention considerations were undertaken, but the patient was informed about the importance of regular visits and follow-up.

## 3 Discussion

Echinococcal infection of the heart is extremely rare finding, and the condition may have passed unnoticed for many years before it may be evident clinically. It can cause various types of cardiac arrhythmias, valvular dysfunctions, pulmonary and systemic embolization, pulmonary hypertension, or many other cardiac or pulmonary presentations [6].

Isolated cardiac involvement may cause no specific symptoms, the patient may have involvement of many other sites simultaneously, which should be excluded by means of imaging studies. The left ventricle is the most common site in the heart involved by the hydatid diseases which is seen in around 60% of the cases of cardiac hydatid disease, followed by the interventricular septum, the right atrium, the right ventricle, the left atrium and the pericardium in order of decreasing frequency [6].

When the cyst rupture into the pericardial space it results in hydatid pericarditis, or it may rupture to the cavity of the right ventricle resulting in pulmonary embolism by the contents of the cyst such as the cyst membrane or the daughter cysts [7].

The diagnosis of cardiac hydatid cyst may be difficult, two dimensional echocardiography or trans-esophageal echocardiography show the cystic lesion and its relations to different cardiac chambers but it may not differentiate hydatid cysts from congenital pericardial cysts. ECG may be quite normal even when the interventricular septum is involved and angiography can differentiate it from ventricular aneurysms [4,8].

CT-scan and MRI can give information about the nature of the cysts and the presence of cysts in other anatomical sites [4,9].

Hydatid cyst should be one of the differential diagnoses in any cystic lesion in all parts of the body especially in patients from endemic areas of history of travelling to the endemic areas. Other differential diagnoses of cardiac hydatid cyst may include pericardial cysts, cardiac tumors, mediastinal tumors, and ventricular aneurysms [6,10].

Surgery is the best options of treatment of cardiac hydatid disease, when the disease is affecting the pericardium complete excision may be possible, but when the myocardium is involved it may be difficult or even impossible to do complete excision, in this situation the cyst contents should be evacuated completely, and it is mandatary to prevent spillage of the contents of the cyst to prevent recurrence. During surgery care must be taken to avoid damage to the conductive system, the papillary muscles, the aortic and the mitral valves. Medical treatment with anthelminthic medications is used after surgery to reduce the recurrence rate [4,6,11].

When the symptoms of the patients don't improve, other possibilities should be listed such as the wrong diagnosis of the possibility of other pathology that may coexist like our patient who had coexisting mitral valve stenosis with hydatid cyst of the right ventricle.

## Patient perspectives

I was very worried about my disease status, having 2 problems in the heart is a major concern, however I had no choice other than surgery. After surgery although I feel much better but I am still worried about the future consequences and the possibility of recurrence.

### Informed consent

An informed written consent was taken from the patient for reporting this case and the accompanying images.

### Provenance and peer review

Not commissioned, externally peer reviewed.

## Ethical approval

No ethical committee approval was needed; consent have been taken from the family to report their findings.

## Sources of funding

No source of funding other than the authors.

## Author contribution

The surgeon who performed the procedure: Dr Alaa S. Bahjat Arif and Dr Ahamd Mohammad Sharif Tahir. Study design, writing, and the final approval of the manuscript: Dr Ayad Ahmad Mohammed.

## Registration unique identifying number

N/A.

## Guarantor

Dr Ayad Ahmad Mohammed.

## Declaration of competing interest

No conflicts of interest present.

## References

[1] Arif S.H., Mohammed A.A. (2018). Primary hydatid cyst of the urinary bladder. BMJ Case Rep..

[2] Thameur M., Habib (2001). Cardiopericardial hydatid cysts. World J. Surg..

[3] Di Bello R. (1967). Hydatid cyst of the left ventricle of the heart: acute hydatid pericarditis∗. Am. J. Cardiol..

[4] Cantoni S. (1993). Hydatid cyst of the interventricular septum of the heart: MR findings. AJR. Am. J. Roentgenol..

[5] Agha R.A. (2018). The SCARE 2018 statement: updating consensus Surgical CAse REport (SCARE) guidelines. Int. J. Surg..

[6] Tetik O. (2002). Giant Hydatid cyst: in the Interventricular septum of a pregnant woman. Tex. Heart Inst. J..

[7] Paşaoğlu I. (1992). Right ventricular hydatid cyst causing recurrent pulmonary emboli. Eur. J. Cardiothorac. Surg: Off. J. Eur. Assoc. Cardio-thoracic Surg..

[8] Rey M. (1991). Diagnostic value of two-dimensional echocardiography in cardiac hydatid disease. Eur. Heart J..

[9] Tahir A.M.S., Bahjat A.S., Mohammed A.A. (2019). Primary infected hydatid cyst of the thigh in a young lady; case report with literature review. Ann. Med. Surg..

[10] Mohammed A.A., Arif S.H. (2019). Hydatid cyst of the calf presenting as painless mass; a case report. Int. J. Surg. Case Rep..

[11] Mohammed A.A., Arif S.H. (2019). Hydatid cyst of the parietal peritoneum. J. Pediatr. Surg. Case Rep..

